# Cytogenetic features of intergeneric amphydiploids and genome-substituted forms of wheat

**DOI:** 10.18699/vjgb-24-80

**Published:** 2024-11

**Authors:** E.D. Badaeva, R.O. Davoyan, N.A. Tereshchenko, E.V. Lyalina, S.A. Zoshchuk, N.P. Goncharov

**Affiliations:** N.I. Vavilov Institute of General Genetics of the Russian Academy of Sciences, Moscow, Russia; National Center of Grain named after P.P. Lukyanenko, Krasnodar, Russia; N.I. Vavilov Institute of General Genetics of the Russian Academy of Sciences, Moscow, Russia; N.I. Vavilov Institute of General Genetics of the Russian Academy of Sciences, Moscow, Russia; Engelhardt Institute of Molecular Biology of the Russian Academy of Sciences, Moscow, Russia; Institute of Cytology and Genetics of the Siberian Branch of the Russian Academy of Sciences, Novosibirsk, Russia

**Keywords:** genome stabilization, wheat, amphydiploid, Aegilops, Dasypyrum, Tritordeum, genome-substituted forms, karyotype, C-banding, fluorescence in situ hybridization, становление геномов, пшеница, амфидиплоиды, Aegilops, Dasypyrum, Tritordeum, геномно-дополненные формы, геномно-замещенные формы, кариотип, С-бэндинг, флуоресцентная in situ гибридизация

## Abstract

Synthetic intergeneric amphydiploids and genome-substituted wheat forms are an important source for transferring agronomically valuable genes from wild species into the common wheat (Triticum aestivum L.) genome. They can be used both in academic research and for breeding purposes as an original material for developing wheat-alien addition and substitution lines followed by translocation induction with the aid of irradiation or nonhomologous chromosome pairing. The chromosome sets and genome constitutions of allopolyploids are usually verified in early hybrid generations, whereas the subsequent fate of these hybrids remains unknown in most cases. Here we analyze karyotypes of five hexa- (2n = 6x = 42) and octoploid (2n = 8x = 56) amphydiploids of wheat with several species of the Aegilops, Haynaldia, and Hordeum genera, and six genome-substituted wheat–Aegilops forms, which were developed over 40 years ago and have been maintained in different gene banks. The analyses involve C-banding and fluorescence in situ hybridization (FISH) with pAs1 and pSc119.2 probes. We have found that most accessions are cytologically stable except for Avrodes (genome BBAASS, a hexaploid genome-substituted hybrid of wheat and Aegilops speltoides), which segregated with respect to chromosome composition after numerous reproductions. Chromosome analysis has not confirmed the presence of the N genome from Ae. uniaristata Vis. in the genome-substituted hybrid Avrotata. Instead, Avrotata carries the D genome. Our study shows that octoploid hybrids, namely AD 7, AD 7147 undergo more complex genome reorganizations as compared to hexaploids: the chromosome number of two presumably octoploid wheat-Aegilops hybrids were reduced to the hexaploid level. Genomes of both forms lost seven chromosome pairs, which represented seven homoeologous groups and derived from different parental subgenomes. Thus, each of the resulting hexaploids carries a synthetic/hybrid genome consisting of a unique combination of chromosomes belonging to different parental subgenomes.

## Abstract (ru)

Синтетические межродовые гибриды (амфидиплоиды) и геномно-замещенные формы пшеницы – важный источник для переноса хозяйственно ценных генов от диких видов в геном Triticum aestivum L. Их используют как для решения теоретических задач, так и в практических целях для получения дополненных или замещенных линий, а также для индукции пшенично-чужеродных транслокаций с помощью облучения или негомологичной конъюгации хромосом. Хромосомный и геномный состав аллополиплоидных форм обычно верифицируется в ранних гибридных поколениях, часто дальнейшая судьба этих гибридов остается неизучен ной. В настоящей работе с помощью методов С-дифференциального окрашивания хромосом по Гимза и флуо ресцентной гибридизации in situ (FISH) с ДНК-зондами pAs1 и pSc119.2 мы провели исследование кариотипов пяти гекса- (2n = 6x = 42) и октаплоидных (2n = 8x = 56) геномно-дополненных амфидиплоидов пшеницы с отдельными видами из родов Aegilops, Haynaldia и Hordeum, а также шести гексаплоидных пшенично-эгилопсных геномно-замещенных форм, полученных более 40 лет назад и поддерживаемых в коллекциях разных научноисследовательских учреждений. Показано, что большинство исследованных форм цитогенетически стабильны, однако Авродес (геном BBAASS) – гексаплоидный геномно-замещенный гибрид пшеницы и Ae. speltoides, расщеплялся по хромосомному составу после многих репродукций. Хромосомный анализ не подтвердил ожидаемого геномного состава геномно-замещенной форма Авротата, у которой вместо заявленного N-генома от Ae. uniaristata Vis. обнаружен D-геном. В данной работе показано, что октаплоидные формы проходят через более сложные преобразования геномов, чем гексаплоидные: в двух исследованных предположительно октаплоидных амфидиплоидах АD 7, АD 7147 произошла редукция числа хромосом до гексаплоидного уровня. У обеих форм были утрачены семь пар хромосом из разных родительских субгеномов, представляющих все семь гомеологических групп. В результате у них сформировался смешанный (гибридный) геном, состоящий из уникальной комбинации хромосом нескольких родительских субгеномов

## Introduction

Common wheat Triticum aestivum L. is one of the most important crops. It ranks third to rice and maize in grain global production (Biodiversity, 2024). It is thought that common wheat arose about 8–10 MY BP in northwestern Iran, near Caspian Sea, as a result of hybridization between a tetraploid wheat and wild goat grass Aegilops tauschii Coss. followed by spontaneous chromosome duplication (Kihara, 1975; Dvořák et al., 1998; Feldman, 2001; Feldman, Levy, 2023). Such crosses might have occurred repeatedly and involve different parental wheat and Aegilops forms growing in the same region (Hirosawa et al., 2004; Luo et al., 2007). In turn, the resulting hexaploid wheats might cross to each other and to other species, thereby extending and enriching the gene pool of the novel crop (Feldman, 2001; Wang et al., 2013).

Common wheat is more flexible than cultivated tetraploid species (Dubcovsky, Dvořák, 2007); therefore, it is better suited to new environment when spreading to new areas. It is also characterized by better adaptability, higher yield, larger grains, and easier threshing as compared to hulled tetraploid wheat (Tadesse et al., 2016). The addition of the D genome from Ae. tauschii conferred grain qualities appropriate for the production of bread, one of the staples in human nutrition. Owing to these advantages, common wheat rapidly penetrated from its center of origin to the neighboring areas; then, to Europe Asia and Africa; and, finally, to North and South America and Australia. It gradually replaced hulled tetra- and hexaploid wheat species. Having been cultivated for over eight millennia, it occupied vast regions with diverse soil and climate conditions.

Meanwhile, intense breeding for high yield, which involved a limited number of founder varieties, narrowed considerably the gene pool of common wheat (Martynov et al., 2006; Girma, 2017; Feldman, Levy, 2023) in the past century. The task of gene pool expansion and search for new donors of commercially valuable traits is increasingly important (Bespalova, 2015). Wild Crop Relatives (WCR) are considered to be among the most promising donors of new genes for wheat improvement (Prohens et al., 2017; Sharma M.P. et al., 2020; Sharma S. et al., 2021). Species of the Aegilops L. genus, wheat relatives, possess many agronomically valuable traits that can be used in wheat breeding: pest resistance, drought tolerance, high micronutrient content, and others (Gill et al., 1986; Monneveux et al., 2000; Schneider et al., 2008; Molnár-Láng et al., 2015; Olivera et al., 2018; Kishii, 2019; Kumar et al., 2019). The close phylogenetic relationship between the Triticum L. and Aegilops genera facilitas successful transfer of genetic material transfer between them, as plasmon and two of the three common wheat nuclear sub-genomes, B and D, have been inherited from Aegilops species (Kihara, 1975; Tsunewaki, 1996).

Nevertheless, the direct gene transfer from Aegilops to wheat is a difficult task. Several approaches have been suggested to improve the efficiency of alien genetic material transfer. One of them involves crosses between wheat and a target species, chromosome doubling in the F1, and developing wheat-alien addition and substitution lines. These lines are then used for inducing wheat-alien translocations (Peng et al., 2011; Zhang P. et al., 2015; Kishii, 2019; Kroupin et al., 2019). For instance, this approach was used to obtain wheat addition and substitution lines with rye (Gill, Kimber, 1974), barley (Islam, Shepherd, 1990; Cabrera et al., 1995; Molnár-Láng et al., 2000; Trubacheeva et al., 2009), Aegilops (Friebe et al., 1992, 2000; Logojan, Molnár-Láng, 2000; Molnár-Láng et al., 2014), Haynaldia villosa (L.) Schur (syn. Dasypyrum villosum (L.) P. Candargy) (Minelli et al., 2005), Thinopyrum Á. Löve (syn. Elytrigia Desv.) (Schulz- Schaeffer, Friebe, 1992; Linc et al., 2012; Kroupin et al., 2019), and other cereals. A number of allopolyploid hybrids between various tetraploid wheat species and Ae. tauschii have been obtained at СIMMYT, Mexico (Kishii, 2019; Aberkane et al., 2020). Pedigree analyses indicate that the genetic material of Aegilops, mainly Ae. tauschii, as well as Ae. umbellulata Zhuk. and Ae. ventricosa Tausch is present in over 1,350 varieties and 9,000 elite lines of common wheat (Martynov et al., 2015), and their ratio is still increasing.

In addition to commercial breeding, synthetic allopolyploids are extensively used in studies of processes accompanying hybrid genome formation (Özkan et al., 2001; Kashkush et al., 2002; Levy, Feldman, 2004). Addition and substitution lines obtained from such allopolyploids were successfully used for the establishing of genetic relationships (homoeology) of chromosomes of different cereal species (Dhaliwal et al., 1990; Cabrera et al., 1995; Friebe et al., 1995a, b, 2000; Badaeva et al., 2018). However, these studies were primarily focused on processes occurring at early stages of allopolyploid formation, whereas their fate remained unknown in most cases.

Another approach was proposed by Dr. E.G. Zhirov. It is based on the development of genome-substituted common wheat forms, in which their D genome is substituted by the genome of a diploid Aegilops or of other cereal species (Zhirov, Ternovskaya, 1984; Davoyan R.O. et al., 2012). The first step of the production of these forms involved the extraction of the tetraploid BBAA component from common wheat cv. Avrora. The resulting tetra-component, tetraAvrora, was crossed to a diploid Aegilops species, whose genome was expected to replace common wheat D genome. The hybrids were treated with colchicine to double the chromosome number and obtain fertile amphydiploids. In spite of the fact that some genome-substituted forms obtained by E.G. Zhirov were cytologically characterized and are still used as donors of resistance genes in the breeding of common wheat and triticale (×Triticosecale Wittm.) (Davoyan R.O., Zhirov, 1995; Davoyan E.R. et al., 2012, 2023; Davoyan R.O. et al., 2019), most of these hybrids have not been analyzed by C-banding.

This article is aimed in cytogenetic verification of intergeneric synthetic amphydiploids and genome-substituted accessions of common wheat obtained over 30 years ago and maintained in gene banks of different institutions using C-banding and (for some hybrids) fluorescence in situ hybridization (FISH).

## Materials and methods

Experiments were conducted with the following artificial genome-substituted hybrids and intergeneric amphydiploids shown in the Table.

**Table 1.**
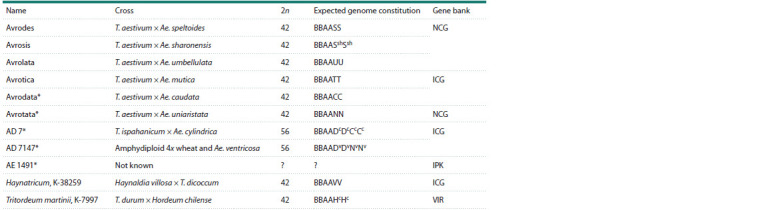
Material examined Notе. * Amphydiploids and genome-substituted accessions with unproved chromosome numbers, genome constitutions, or chromosome sets. NCG – National Center of Grain named after P.P. Lukyanenko, Krasnodar, Russia; ICG – Institute of Cytology and Genetics of the Siberian Branch of the Russian Academy of Sciences, Novosibirsk, Russia; IPK – Leibniz-Institut für Pflanzengenetik und Kulturpflanzenforschung, Gatersleben, Germany; VIR – N.I. Vavilov All-Russian Institute of Plant Genetic Resources (VIR), St. Petersburg, Russia.

Six genome-substituted forms were raised by Dr. E.G. Zhirov at the Lukyanenko Research Institute of Agriculture, Krasnodar, more than 40 years ago. Their detailed description is provided in Zhirov’s Dr. Sci. thesis “Wheat genomes: study and reconstruction” (Kyiv, Institute of Plant Physiology and Genetics, National Academy of Sciences, Ukraine, 1989). Two wheat–Aegilops amphydiploids were obtained by G.B. Piralov (1976) at the Institute of Genetics and Breeding, Academy of Sciences of the Azerbaijan SSR, Baku. One was accidently found in the collection of the Institute of Cultivated Plants, IPK, Gatersleben, Germany. Its origin is unknown. The hybrid between emmer and Haynaldia villosa was synthesized by P.M. Zhukovsky (1944). The amphydiploid of durum wheat and wild barley Hordeum chilense Roem. & Schult. was developed in Spain in the early 1980s (Martin, Sanchez-Mongelaguna, 1982; Fernández, Jouve, 1984).

Karyotypes were analyzed by the conventional Giemsa C-banding protocol (Badaeva et al., 1994). Tritordeum was additionally analyzed by FISH (Badaeva et al., 2017) with DNA probes pAs1 (Rayburn, Gill, 1986) and pSc119.2 (Bedbrook et al., 1980). Wheat chromosomes were identified according to B.S. Gill et al. (1991), and chromosomes of other species, according to the nomenclatures proposed in (Dhaliwal et al., 1990; Cabrera et al., 1995; Friebe et al., 1995а, 2000; Linc et al., 1999; Badaeva et al., 2008, 2011, 2015a; Liu et al., 2010; Adonina et al., 2015; Molnár et al., 2016; Danilova et al., 2017; Said et al., 2021).

## Results and discussion

Genome-substituted forms

Avrodes

**Fig. 1.**
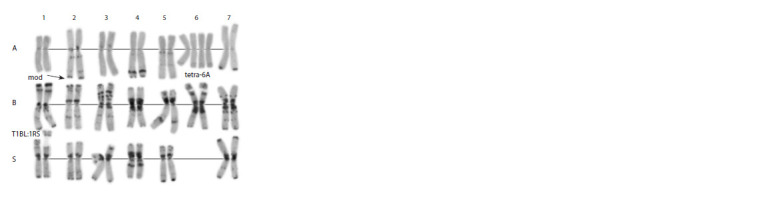
C-banding karyotype of the Avrodes genome-substituted form. A, B, S – genomes; 1–7 – homoeologous groups.

**Fig. 2.**
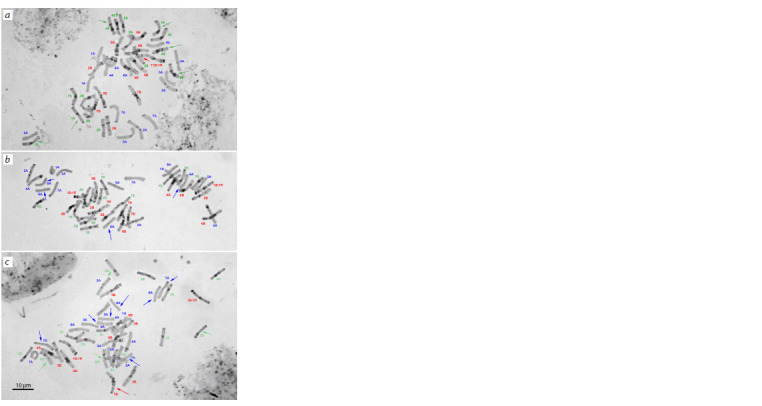
C-banded metaphase plates in plants of the Avrodes genome-substituted form with different chromosome combinations. Arrows indicate mono-, tri-, and tetrasomic chromosomes belonging to different genomes: red arrows – B genome; blue – A; and green – S.

Avrodes was cytogenetically proven to be hexaploid form in which the D genome is replaced by genome S from Ae. speltoides Tausch (Figs. 1, 2). Avrodes is cytologically unstable, and its chromosome numbers and combinations of the A, B, and S genome chromosomes vary among individual plants

The plants examined had seven or eight A genome chromosome pairs. All plants had 1А, 2А, 4А, 5А, 6А, and 7А. Chromosome 2A of Avrodes differed from 2A of Avrora in having clear telomeric and terminal C bands. Unlike other chromosomes of the A genome, 6A was present as tetrasome, one pair substituting 6S. Most Avrodes plants had only one 7A pair, but two had an additional copy, substituting 7B (monosomic 7A/7B substitution; Fig. 2c). The karyotype of one plant lacked chromosome 3A, which had been replaced by an additional 3S pair.

Only three of seven chromosomes of the B genome, namely, 2B, 3B and 6B were present in all Avrodes plants examined. The 1BL:1RS wheat–rye translocation inherited from Avrora was seen in all plants, but the translocated chromosome was present in one or two copies (monosomic 1BL:1RS/1S substitution), or it was modified by a translocation of an unidentified fragment onto the distal portion of the long 1B arm (Fig. 2a, red arrow).

**Fig. 3.**
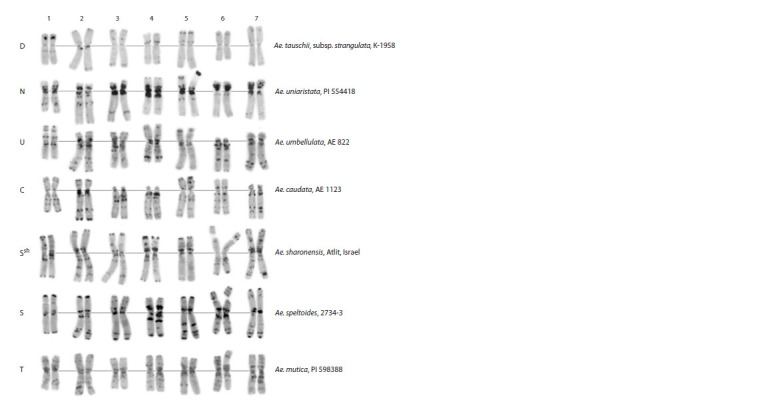
C-banded karyotypes of diploid Aegilops species supposedly or actually involved in the development of genome- substituted wheat forms. “Type specimens” of species not involved in the development of forms examined are shown for reference. The D–T genome symbols are indicated on the left; species names and origin/accession vouchers, on the right.

Some plants were nulli4B-tetra4S (Figs. 2a, b) and others, nulli5B-tetra4S (Figs. 2a, b), where the two 5S chromosome pairs showed different C-banding patterns (Fig. 2c, green arrows). One pair matched exactly chromosome 5S of Ae. speltoides, and the other, designated as 5S*, was shorter, and it lacked the large telomeric band in the long arm (Fig. 3). Note that just this modified chromosome pair passed to Avrodes-derived elite accessions resistant to stripe or yellow rust (Puccinia striiformis Westend. f. sр. tritici Eriks.) (Davoyan E.R. et al., 2023).

The S genomes of different Avrodes plants was represented by 12 to 16 chromosomes, but chromosome 6S was always missing (Fig. 2). Only 2S and 7S were present only in the disomic state. Chromosomes 3S, 4S, and 5S were present as di- or tetrasomics, where 3S replaced homoeologs of the A genome, and 4S and 5S, of the B genome. Most plants had one 1S pair, and only two had an additional chromosome 1S, which replaced 1BL:1RS (monosomic 1S/1BL:1RS substitution, Fig. 2a).

The significant cytological instability of Avrodes also manifested itself in an abnormal meiotic chromosome pairing, in particular, high frequency of multivalents, formerly reported by R.O. Davoyan et al. (2012, 2019). This high frequency may be due to both the presence of genes suppressing Ph1 (the gene regulating homoeologous chromosome pairing) in the S genome (Dvořák et al., 2006), and occurrence of intergenomic B/S or A/S substitutions in most plants, which have three or four copies of some Ae. speltoides chromosomes. The genomic instability of Avrodes may also be contributed by gametocidal genes, located on chromosomes 2S and 6S in Ae. speltoides (Tsujimoto, Tsunewaki, 1988; King J. et al., 2018; Said et al., 2024). It is worth noting that we found only one of the gametocidal chromosomes, 2S, whereas 6S had been lost.

Avrosis

**Fig. 4.**
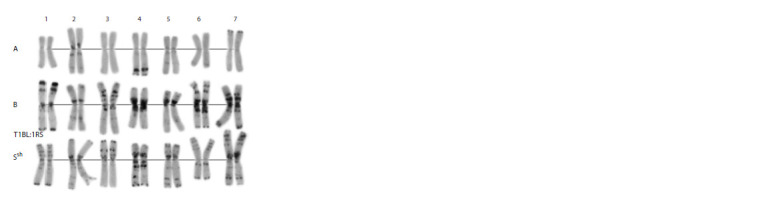
Karyotype of the genome-substituted form Avrosis. A, B, Ssh – genomes; 1–7 – homologous groups.

Avrosis is a hexaploid in which the D genome is replaced by the Ssh genome from Ae. sharonensis. Eig. The presence of the A, B, and Ssh genomes was proven by cytogenetic analysis, including C-banding (Fig. 4). Like Avrodes, Avrosis bears the 1BL:1RS wheat–rye translocation. However, the C-banding patterns of 2A, 2B, 3B, and 5B chromosomes of these forms differed from each other. Unlike Avrodes, Avrosis is cytologically stable: all plants examined had identical chromosome composition and C-banding patterns.

Chromosome T1B:1R was the only exception. In some plants, the distal portion of the short arm was deleted. The chromosomes of the Ssh genome showed the morphology and heterochromatin distribution typical of Ae. sharonensis (Fig. 4). However, the direct parental accessions of Avrosis had not been indicated by originator, and we could not reveal chromosome changes associated with polyploidization.

In contrast to Avrodes, Avrosis was used in breeding programs solely as a donor of powdery mildew (Blumeria graminis (DC.) Speer f. sр. tritici Marshal) resistance (Zhirov, Ternovskaya, 1993), although Ae. sharonensis possesses many agronomically valuable traits (Olivera, Steffenson, 2009; Millet et al., 2014). The main difficulty in Avrosis use is that the Ssh genome hosts highly efficient gametocidal genes Gc (Tsujimoto, Tsunewaki, 1984, 1988; Said et al., 2024). They induce the lethality of gametes that have lost the 4Ssh chromosome, bearing this gene. As a result, the 4Ssh chromosome is preferentially transmitted to gametes (Miller et al., 1982; King I. et al., 1991).

Nevertheless, some scientists succeeded in obtaining wheat × Ae. sharonensis introgression lines for chromosomes of other homoeologous groups, in particular, 1Ssh and 5Ssh (Millet et al., 2014). considering these results, we can hope that other Ssh chromosomes can be transmitted to the progeny and the genetic potential of Avrodes in common wheat breeding is far from being exhausted.

Avrolata

**Fig. 5.**
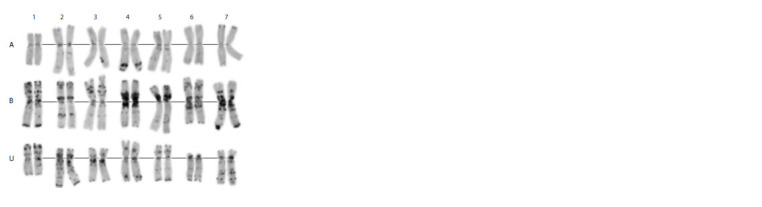
Karyotype of the genome-substituted form Avrolata. A, B, U – genomes; 1–7 – homologous groups.

Avrolata is a hexaploid wheat in which the D genome is replaced by the U genome of Ae. umbellulata. It is cytologically stable, like Avrosis. All its plants had identical genome constitutions and banding patterns. We found no chromosome rearrangements in the accession studied. C-banding analysis confirmed the presence of the A, B, and U genomes in its karyotype (Fig. 5). In contrast to Avrodes and Avrosis, Avrolata did not bear the 1BL:1RS wheat–rye translocation; rather, it had the normal 1B chromosome.

The C-banding patterns of chromosomes belonging to the A and B genomes were generally similar to those of Avrosis, and the U chromosomes showed morphologies and banding patterns typical of Ae. umbellulata (Figs. 3, 5). As the parental Ae. umbellulata form was unknown, we could not assess putative changes of the U genome chromosomes of this hybrid.

The lack of 1BL:1RS in the karyotype of Avrolata may be due to the fact that cv. Avrora was ab initio heterogeneous for the presence of this translocation, and the direct parent of Avrolata belonged to the biotype lacking it. It is conceivable that durum wheat chromosome 1B survived recurrent crosses in the extraction of the Avrora tetra-component

As reported in (Davoyan E.R. et al., 2012; Davoyan R.O. et al., 2012), Avrolata, along with Avrodes, is a source of novel genes for leaf rust (Puccinia triticina Rob. ex Desm. f. sр. tritici Eriks.) resistance. It is known that Ae. umbellulata, which was the source of the U genome in Avrolata, is extensively used in common wheat breeding, especially in the United States, as donor of the Lr9 leaf rust resistance gene (Friebe et al., 1996b; McIntosh et al., 2013). Pedigree analysis shows that the ratio of varieties obtained with the use of Ae. umbellulata constantly increases and constitutes 25–29 % in 2000s (Martynov et al., 2015). Although Lr9 had been detected in Avrolata, it was not found in its progeny (Davoyan E.R. et al., 2012). Apparently, the resistance in the derived accessions was determined by a novel Lr gene or a couple of unidentified genes. Avrolata was also employed in the breeding of other crops. A molecular study demonstrated the transmission of chromosomes 1U and 2U to the progeny of Avrolata crosses with winter hexaploid triticale (Orlovskaya et al., 2015).

Avrotica

Avrotica is a genome-substituted form, whose parents were common wheat cv. Avrora and Ae. mutica Boiss. (syn. Amblyopyrum muticum (Boiss.) Eig, Т genome). Cytogenetic analysis proved that Avrotica bears chromosomes of wheat A and B genomes and the T genome of Ae. mutica. However, in contrast to previously considered genome-substituted forms, Avrotica has a more complex combination of parental chromosomes.

**Fig. 6.**
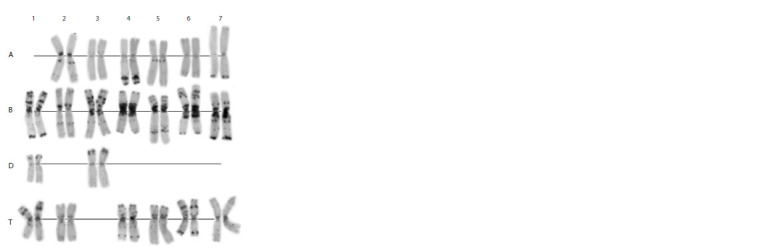
Karyotype of the genome-substituted form Avrotica A, B, D, T – genomes; 1–7 – homoeologous groups.

Specifically, its karyotype maintained two chromosomes of the D genome, 1D and 3D, but lacked wheat 1A and Ae. mutica 3T (Fig. 6). Thus, the alien genome is represented by only six chromosome pairs. Like Avrolata, Avrotica did not possess the wheat–rye 1BL:1RS translocation, although the C-banding patterns of other chromosomes were similar to those of Avrodes. We could not compare T chromosomes with those of the parental Ae. mutica accession, because the originators had not indicated the source of the latter. It should be noted that the homologous T chromosomes of the amphydiploid showed identical banding patterns, whereas the diploid species is highly polymorphic; in particular, is characterized by heteromorphism of homologs (Friebe et al., 1996a).

Although Avrotica is found to be rust resistant (Davoyan R.O. et al., 2012, 2019), this trait has not been transferred to common wheat. A Chinese team (Liu et al., 2015) produced a powdery mildew resistant incomplete amphydiploid of cv. Chinese Spring with Ae. mutica and an addition line for chromosome 7T. The allopolyploid had the complete set of the T genome chromosomes but lacked the pair of wheat chromosome 7B.

Another team crossed common wheat cvs. Chinese Spring and Pavon 76 to Ae. mutica accession bearing genes – suppressors of the Ph1 locus (King J. et al., 2017). The F1 hybrids were twice or thrice backcrossed to the parental cultivar. The plants were scored for alien introgressions by SNP genotyping. Genotypes with single introgressions were used to produce di-haploid plants. This procedure yielded 67 homozygous and stably inheritable introgression lines involving six of the seven Ae. mutica chromosomes (King J. et al., 2019). The team failed to obtain introgression lines for chromosome 3T, which was absent from Avrotica as well. It is reasonable to conjecture that this chromosome bears genes adversely affecting the viability and/or fertility of the T. aestivum × Ae. mutica allopolyploid; for this reason, plants carrying 3T were abandoned by selection in early hybrid generations.

Avrodata

**Fig. 7.**
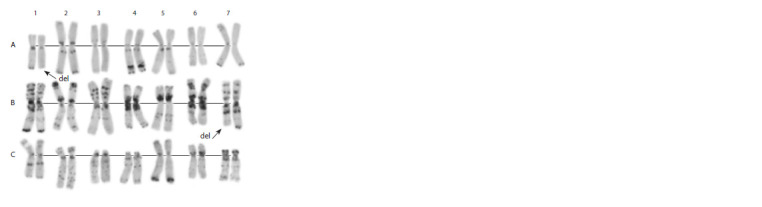
Karyotype of the genome-substituted form Avrodata. A, B, C – genomes; 1–7 – homoeologous groups. The arrow indicates a terminal deletion/translocation involving long arms of 1A and 7B chromosomes.

The pedigree of Avrodata indicates that it was obtained by crossing common wheat Avrora and Ae. caudata L. (syn. Ae. markgrafii (Greuter) Hammer). Cytological analysis confirmed the presence of the A and B wheat genomes and the C genome of Ae. caudata (Figs. 3, 7). All plants examined had identical chromosome sets, but chromosome rearrangements were detected in some of them (Fig. 7). They may have been induced in wheat–Ae. caudata crosses by gametocidal genes located on chromosome 3C (Endo, Tsunewaki, 1975).

The Avrodata lacks the 1BL:1RS wheat–rye translocation, and C-banding patterns of most chromosomes of the A and B wheat genomes (e. g., 2А, 4А, 5А, 6А, 1В, 2В, 5В, 6В, 7В) differed from the corresponding chromosomes of other genome-substituted forms obtained with cv. Avrora. In particular, the banding pattern of chromosome 7B was more similar to 7B of durum rather than common wheat. These observations suggest that Avrodata had been obtained from another parental wheat form or that the extraction of the tetra-component from Avrora resulted in the transmission of only part of A and B genome chromosomes of common wheat. The presence of unbalanced chromosome rearrangements in Avrodata plants shows that this genome-substituted form is cytologically unstable. No information on the use of this accession in breeding has been reported.

A genome-substituted amphydiploid of common wheat cv. Alcedo and Ae. caudata was synthesized in Germany (Blüthner et al., 1988). The octoploid amphydiploid and addition lines developed on its basis were analyzed by C- banding, and meiotic chromosome pairing was also studied (Blüthner et al., 1988; Friebe et al., 1992). No deviations in C-banding patterns caused by chromosome rearrangements were detected, although numerous aberrations were noted in meiosis in all studied lines (Friebe et al., 1992). The banding pattern deviations observed in some wheat chromosomes were attributed to putative involvement of other wheat varieties in its origin

The poor use of Avrodata in breeding may be due to the difficulty of the transmission of C genome material to common wheat associated with (1) a large number of speciesspecific chromosome rearrangements found in Ae. caudata (Danilova et al., 2017; Gong et al., 2017; Grewal et al., 2020) and (2) the presence of gametocidal genes on Ae. caudata chromosomes.

Avrotata

**Fig. 8.**
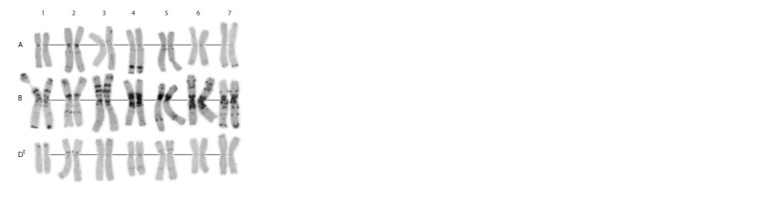
Karyotype of the genome-substituted form Avrotata. A, B, Dt – genomes; 1–7 – homoeologous groups.

We found that Avrotata is a cytologically stable hexaploid form. Its karyotype contains the A and B wheat genomes but no chromosomes corresponding to the N genome of Ae. uniaristata Vis have been detected (Figs. 3, 8). The third Avrotata genome showed the greatest similarity to the D genome of diploid Ae. tauschii subsp. strangulata Eig. (Fig. 3), which differs from the wheat D genome in C- banding patterns of chromosomes 3D and 6D. Presumably, the third Avrotata genome, Dt, is a mix of chromosomes derived from diploid Ae. tauschii and the D genome of common wheat, but this assumption cannot be proven by C-banding, because orthologous chromosomes of these genomes are closely similar.

Ae. uniaristata is tolerant to aluminum, and British scientists synthesized a hybrid between Chinese Spring and Ae. uniaristata to transmit this trait to the common wheat genome. This hybrid was employed in the development of several addition lines (Miller et al., 1995). The scientists showed that aluminum tolerance is controlled by chromosome 3N (Iqbal et al., 2000b). Analyses of the lines by in situ hybridization (Iqbal et al., 2000a) and later by C-banding (Badaeva et al., 2011) confirmed that they bear Ae. uniaristata chromosomes. These data allowed the cytological and genetic classifications of chromosomes of the N genome to be brought into compliance.

The mapping of RFLP markers on Ae. uniaristata chromosomes showed that they had been considerably rearranged with regard to homoeologous wheat chromosomes owing to the N genome-specific translocations and inversions (Iqbal et al., 2000b). The deep structural rearrangements of Ae. uniaristata chromosomes over the course of speciation were confirmed by the results of chromosome painting with oligo probe cocktail specific to each of the seven homoeologous groups of Triticeae (Li et al., 2020). It is reasonable to assume that the divergence of homoeologous wheat and Ae. uniaristata chromosomes impedes the transfer of genetic material between species, including the development of stable viable amphydiploids and genome-substituted forms. Unfortunately, no available data on the cytological verification of the genome constitution of Avrotata during early stages of its development have been reported. For this reason, we cannot decide whether the absence of the N genome from Avrotata was determined by the difficulty in the development of the form itself or the D genome replaced the N over the course of material propagation.

## Wheat–Aegilops amphydiploids

Amphydiploid AD 7

AD 7 is a spontaneous amphydiploid of tetraploid wheat T. ispahanicum Heslot (genome BBAA) and tetraploid Ae. cylindrica Host (DcDcCcCc). The ancestral form of AD 7 was an octoploid 2n = 8x = 56 with the genome constitution BBAADcDcCcCc (Mustafaev, Piralov, 1981). C-banding analysis confirmed the origin of the accession from Ae. cylindrica but showed that chromosome number of amphydiploid was reduced to hexaploid level.

**Fig. 9.**
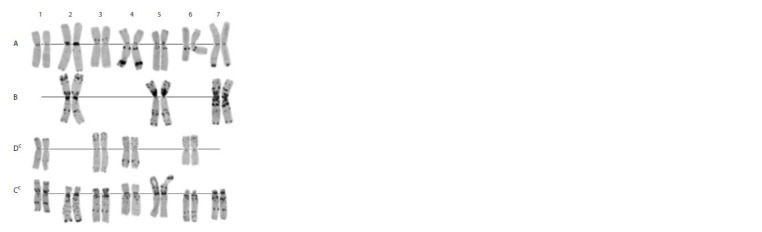
Karyotype of the AD 7 amphydiploid A, B, Dc, Cc – genomes; 1–7 – homoeologous groups

Complete set of the wheat A-genome and Ae. cylindrica Cc-genome chromosomes were preserved in AD 7. The third genome proved to be mixed. It combined chromosomes of the wheat B genome and Ae. cylindrica Dc genome, so that all the seven homologous groups were represented: 1Dc1Dc 2B2B 3Dc3Dc 4Dc4Dc 5B5B 6Dc6Dc 7B7B (Fig. 9). The chromosomes of the Dc genome showed banding patterns typical of Ae. cylindrica (Linc et al., 1999; Badaeva et al. 2002). Some plants were monosomic for chromosome 6A (2n = 41). No chromosome rearrangements were found in the plants studied.

Amphydiploid AD 7147

**Fig. 10.**
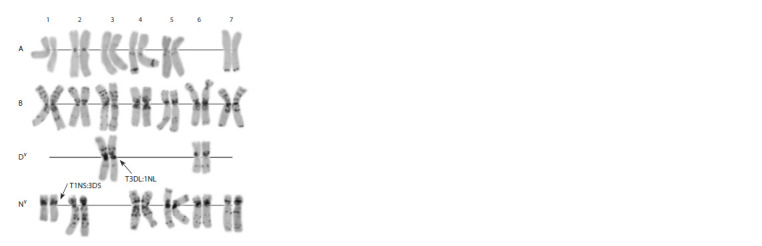
Karyotype of the AD 7147 amphydiploid. A, B, Dv, Nv – genomes; 1–7 – homologous groups.

Amphydiploid AD 7147 was obtained by G.R. Piralov (1976) by crossing tetraploid wheat and Ae. ventricosa (Mustafaev, Piralov, 1981). The chromosome number doubled spontaneously; as supposed by G.R. Piralov, owing to the fusion of unreduced gametes. Regular chromosome pairing yielding 28 bivalents was observed in the meiosis of the original 56-chromosome amphydiploid. C-banding analysis of the AD 7147 confirmed that its origin from tetraploid wheat (genome BBAA) and Ae. ventricosa (genome DvDvNvNv) (Fig. 10). However, the C-banding patterns of the A and B genome chromosomes differed from those typical of durum wheat, being closer to T. carthlicum Nevski or the European variety of emmer T. dicoccum Schrank ex Schübl. (Badaeva et al., 2015b).

We found that AD 7147 bears a 1Nv:3Dv translocation, most likely, inherited from the parental Aegilops accession. This translocation is common in natural Ae. ventricosa populations (Badaeva et al., 2002, 2011). As in the previous amphydiploid, the chromosome number in AD 7147 was reduced to hexaploid level as a result of a loss of one “hybrid” genome. In this case, though, the wheat B genome remained intact, 3Nv was lost from the Nv genome, and 6A, from the wheat A genome. Thus, the chromosome number reduction in the hybrid involved mainly the Dv genome of Ae. ventricosa, of which only two chromosome pairs were preserved: 3Dv (in the form of two translocated chromosomes T1Nv:3Dv) and 6Dv.

Ae. ventricosa is tetraploid species. Presently, it is extensively employed in wheat breeding as donor of pest resistance genes (Dosba, Doussinault, 1978; Garcia-Olmedo et al., 1984; Delibes et al., 1987, 1988). The gene cluster Sr38/Lr37/Yr17, inherited from Ae. ventricosa (Tanguy et al., 2005), had been mapped on chromosome 2A (Bariana, McIntosh, 1994). Pedigree analysis (Martynov et al. 2015) shows that this introgression is present in more than 34–37 % of modern common wheat varieties, mostly of European origin. The introgression originates from the French VPM- 1 breeding line, which was produced by Maia in 1967 by crossing common wheat cv. Marne and a synthetic amphydiploid Ae. ventricosa × T. persicum Vav. (syn. T. carthlicum Nevski) (Dosba et al., 1978). Apparently, the genome constitution of this amphydiploid is similar to that of the original AD 7147 accession, but we cannot test the modern constitution of the French hybrid. We have no information on the use of AD 7146 in wheat breeding either, but, by way of analogy with the French Ae. ventricosa × T. persicum, it could be a promising donor of agronomically important genes.

Amphydiploid AE 1491

**Fig. 11.**
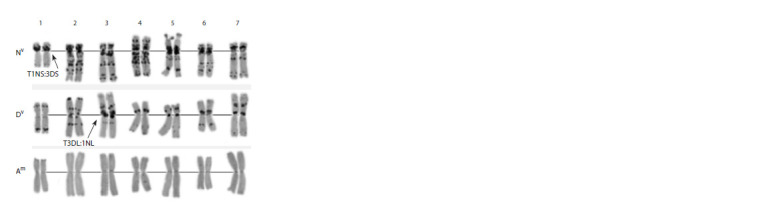
Karyotype of the AE 1491 amphydiploid. Nv, Dv, Am – genomes; 1–7 – homologous groups.

Synthetic hexaploid amphydiploid AE 1491 was accidently identified among Aegilops accessions from the gene bank of the Leibniz Institute of Plant Genetics and Crop Plant Research (Germany). Analysis of chromosome morphology and the C-banding patterns (Fig. 11) brought us to suggestion that it is a hybrid of tetraploid Ae. ventricosa (genome DvDvNvNv) and einkorn wheat, presumably, T. boeoticum Boiss. (genome AbAb) or T. monococcum L. (AmAm). AE 1491 carried a 1Nv:3Dv translocation, and it is conceivable that it was also present in the parental Ae. ventricosa accession.

No cases of aneuploidy, significant changes of C-banding patterns in comparison to the parental species (Badaeva et al., 2002, 2015a), or new variants of chromosome structural rearrangements were detected. An amphydiploid T. aegilopoides Link (syn. T. boeoticum) × Ae. ventricosa was produced and studied by (Siddiqui 2009; Siddiqui et al., 2009), but we do not know whether it corresponds to our accession.

Wheat amphydiploids

Haynatricum Zhuk.

Amphydiploids of wheat and Dasypyrum villosum (syn. Haynaldia villosa) were successfully produced by scientists from different countries starting from the 19th–early 20th century. Crosses to various wheat species, mostly tetraploids (T. dicoccoides (Körn. ex Asch. &Graebn.) Schweinf., T. dicoccum, T. turgidum L., T. aethiopicum Jakubz., T. durum Desf., T. araraticum Jakubz., T. timopheevii (Zhuk.) Zhuk.) or, less often, hexaploids (spelt and common wheat) (Pace et al. 2011) were undertaken. Our T. dicoccum × D. villosum amphydiploid has been developed by P.M. Zhukovsky and named Haynatricum Zhuk. (syn. Triticum ×turgidovillosum Tschermak) (Zhukovsky, 1944). It is maintained in the VIR gene bank under accession number K-38259.

**Fig. 12.**
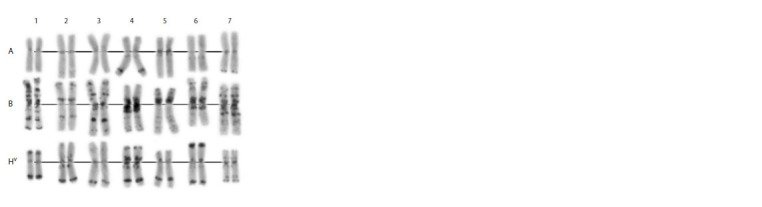
Karyotype of Haynatricum. A, B, Hv – genomes; 1–7 – homoeologous groups.

The accession was shown to bear the entire sets of wheat A and B genome chromosomes and the Hv genome chromosomes of D. villosum (Fig. 12). The C-banding patterns of wheat chromosomes were similar to those of the Transcaucasian group of cultivated emmer (Badaeva et al. 2015). It is likely that the parental form of this allopolyploid was T. dicoccum accession from Armenia, Azerbaijan, or neighboring regions of Turkey or Iran. All Haynatricum plants examined were euploid (2n = 6x = 42). No chromosomal rearrangements were detected. This fact, along with the absence of notable C-banding changes, points to a high cytological stability of the accession, which was obtained nearly 85 years ago.

D. villosum is a good donor of genes for disease resistance. Its amphydiploids and substitution and addition lines derived therefrom are broadly used in wheat breeding in China (Huang et al., 2007; Zhang W. et al., 2013) and other countries. Our accession differs from them in the distribution of heterochromatin blocks on chromosomes of wheat and D. villosum and therefore it is genetically different and may contain a different set of resistance genes.

Tritordeum martinii A. Pujadas

The amphydiploid of durum wheat and wild barley H. chilense was synthesized in the early 1980s as a bridge for transferring agronomically useful genes from barley to wheat (Martin, Sanchez-Mongelaguna, 1982). Its karyotype was examined in detail by C-banding (Cabrera et al., 1995) and FISH with various DNA probes (Prieto et al., 2004; Martín, Cabrera 2005).

**Fig. 13.**
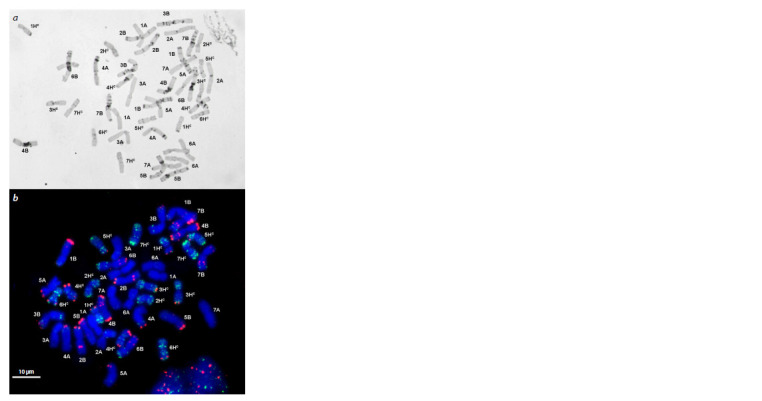
C-banded metaphase plate (a) and the distribution of probes pAs1 (green) and pSc119.2 (red) on Tritordeum chromosomes (b). Chromosome designations: 1A–7A – wheat A genome; 1B–7B – wheat B genome; Hc – H. chilense genome.

Analyses of Tritordeum chromosomes by C-banding (Fig. 13a) and FISH with pAs1 (green) and pSc119.2 (red) (Fig. 13b) probes confirmed the presence of the A, B, and Hc genomes. Their C-banding and FISH patterns did not differ from those described in the literature. No aneuploidy or chromosome rearrangements were detected, which pointed to a good cytological stability of the accession.

## Conclusion

This feature is of great importance for the preservation and propagation of the allopolyploid, which is presently considered to be a new promising man-made crop (De Caro et al. 2024).

The results of the study of genome-substituted and synthetic genome-added amphydiploids of wheat and species of the Aegilops, Dasypyrum, and Hordeum genera bring us to the conclusions that:

The chromosome sets of allopolyploids having 42 chromosomes are more stable than those of octoploids; however,

Hexaploid forms containing related genomes (B–S and Avrodes) can remain cytologically unstable over many generations. The cytological instability manifests itself in the heterogeneity of the chromosome sets of plants wheats may provide new genes for resistance to biotic (Goncharov et al., 2020) and abiotic (Mahmood et al., 2023) stress factors for improving cultivar of common wheat.

## Conflict of interest

The authors declare no conflict of interest.
